# Dye Laser to Treat an Arteriovenous Malformation of the Tongue: 40-Month Follow-Up

**DOI:** 10.1155/2023/5583749

**Published:** 2023-10-11

**Authors:** Niccolò Giuseppe Armogida, Luigi Esposito, Elena Calabria, Mariangela Cernera, Gianrico Spagnuolo, Flavia Iaculli

**Affiliations:** ^1^Department of Neuroscience, Reproductive Sciences and Dentistry, University of Naples Federico II, Naples, Italy; ^2^Department of Health Scientist, University Magna Graecia of Catanzaro, Catanzaro, Italy

## Abstract

Arteriovenous malformations (AVMs) are abnormal connections between blood vessels that bypass the normal capillary bed. To avoid the invasiveness of the gold standard surgical excision, the use of dye laser has been suggested as an alternative. A 53-year-old man in good overall health presented with a large bluish-red nodular growth covered by intact mucosa on the left side of his tongue. The growth had a hard-elastic consistency and was not painful to touch. Imaging investigations revealed a capsulated growth consistent with a diagnosis of AVM. The patient underwent two sessions of rhodamine dye laser treatment using the following parameters: fluence of 12 J/cm^2^, 6 mm laser spot, a single pulse with repetition up to 1.0 Hz, and a pulse duration of 3.0 ms. Follow-up examinations were conducted at 12, 24, 36, and 40 months after the treatment. At the 40-month follow-up, the lesion had reduced in size, with a more organized vascular network, and was not clinically detectable. Considering the limitations of this case report, the application of dye laser appears to be a potentially successful treatment option for AVMs.

## 1. Introduction

Arteriovenous malformations (AVMs) are defined as rare abnormal communication between arteries and veins that bypass the capillary bed, creating a vascular tangle called “nidus.” They are included in the newest classification of vascular anomalies published by the International Society for the Study of Vascular Anomalies (ISSVA) [1].

In 40% of patients, AVMs occur at early age; however, they often become clinically evident later in life. AVMs are most frequently localized in the head and neck region (70%), and they are classified according to Schobinger's staging to determinate their clinical severity [2, 3] and according to Cho et al. [4] and Yakes and Baumgartner [5] to classify the hemodynamic structure.

AVMs represent an important medical and social issue as disabling pathologies that occur with serious functional, aesthetic, and psychological alterations; they also may require surgical extirpation that could be extremely challenging, due to the risk of massive life-threatening intraoperative bleeding and to the need of normal tissue replacement with disease vessels [6].

In this regard, lasers have been proposed for the treatment as a valid noninvasive alternative for the treatment of for vascular anomalies. The Nd:YAG laser employs a 1% neodymium-doped yttrium-aluminum-garnet crystal as its active medium, emitting light at 1,064 nm. Its affinity for hemoglobin results in endothelial thermal effects. It effectively penetrates tissue to depths of 4-7 mm, making it suitable for vascular mucosal lesions. Both 532 nm and 1064 nm Nd:YAG lasers treat vascular and pigmented lesions successfully [7–10]. The diode laser is preferred for excising proliferating lesions, reducing bleeding, and postoperative complications (edema, superinfections, and scars) [11–12]. Typically, it operates at 980 nm, using defocused irradiation mode at 3 watts until the lesion changes color from dark purple to light gray [13]. This laser therapy offers a comfortable alternative to sclerotherapy for oral vascular malformations and is effective for common vascular lesions, with endorsements for both 980 nm and 1470 nm diode lasers [14–15].

To the best of our knowledge, dye laser has never been used for the treatment of oral AVMs.

Therefore, the aim of the present study was to describe a successful case of AVM of the tongue treated with rhodamine dye laser that was able to coagulate both capillaries and red vascular malformations, without involving the superficial epithelial layers, in order to avoid the invasiveness of the gold standard surgical excision or embolization [16].

## 2. Methods

### 2.1. Case Study

**Figure 1:**
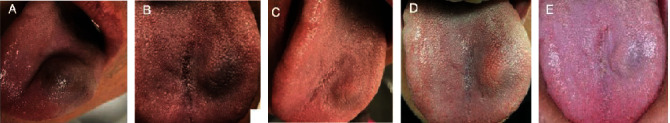
Clinical appearance of the AVM: (A) preoperative optical findings; (B) optical findings at 1-month follow-up and immediately before the second laser session; (C) 12-month follow-up; (D) 24-month follow-up and after the injection of the triamcinolone acetonide; (E) 40-month follow-up.

**Figure 2:**
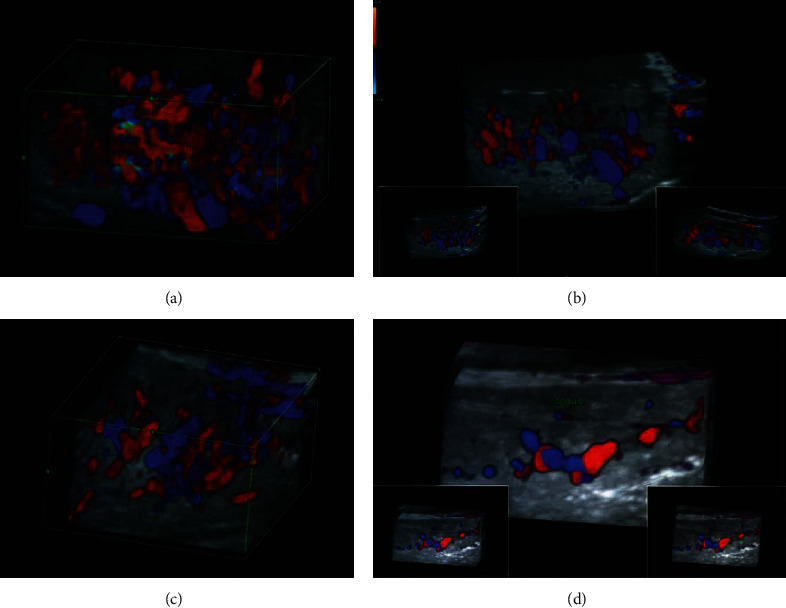
Color/power Doppler imaging of the AVM: (a) baseline; (b) 12-month follow-up; (c) 24-month follow-up; (d) 40-month follow-up.

A male patient aged 53 years, with a good general health and affected for 2 years of a voluminous vascular lesion of the tongue, was referred at the Department of Neuroscience, Reproductive Sciences and Dentistry–University of Naples Federico II. The subject signed a written informed consent, and the study was conducted in accordance with the Declaration of Helsinki. The study was notified to the ethical committee of the University of Naples Federico II. The patient reported difficulties with swallowing and speech, as well as slight pain, secondary to an extensive nodular neoformation involving the left hemitongue in almost all thickness. This vascular lesion had an expansion of 3.5 cm × 2.5 cm. On clinical observation, the nodule was covered by a structurally normal mucosa both on dorsal and ventral surfaces of the interested hemitongue, but with a slightly hyperemic red-blue color (Figure 1(a)). The lesion had a hard-elastic consistency, and it was nonpainful on superficial and deep palpation. According to the Guidelines for Vascular Anomalies draw up by the Italian Society for the study of Vascular Anomalies (SISAV) [16], the ultrasound was performed, in traditional technique, with 3D acquisition and, in multiple acquisition, with TUI (tomographic ultrasound imaging) technique. Color/power Doppler and 3D angio were performed to evaluate the vascularity. Thus, it was possible to define an ovoid-shaped formation, capsulated, immediately under the lingual mucosa, which compressed and dislocated contiguous muscle bundles. The formation had a multivacuolar internal structure with thin septa of uneven thickness and rather disordered texture. The major septa were intensely prelude with mainly arterior signals, supplied by deep branches of lingual arteries (Figure 2(a)). The formation size was 18.2 × 8.8 × 12.2 mm. The lesion was diagnosed as an AVM classified as stage 2 according to Schobinger's staging [2], type IIIb according to Cho classification [4], and type II according to Yakes classification [5]. Patient refused to undergo lesion excision with a hemitongue resection, due to surgical-related complications and unacceptable dysfunction that would ensue. Therefore, application of rhodamine dye laser (595 nm, Synchro VasQ, M.E.L.A s.r.l., Calenzano (FI), Italy) was performed. The technique consisted of irradiation with a rhodamine dye laser with noncontact technique at a distance of 3 cm from the dorsal tongue surface. The laser-induced coagulation was performed in a repetitive manner with the handpiece of the irradiation delivery system perpendicular to the mucosa and lesion as focal point; then, laser energy was delivered to all areas of the vascular malformation. The dye laser was settled with the following operating parameters: fluence at 12 J/cm^2^, handpiece with 6 mm spot, single pulse with repetition up to 1.0 Hz, and pulse duration of 3.0 ms. Patient underwent a local anaesthesia with a perilesional injection with mepivacaine and adrenaline (1 : 100000). The patient and the operator wore protective eye coverings. No recommendation was given to the patient about avoiding foods or beverages. The patient was suggested to take nonsteroidal anti-inflammatory drugs (NSAID), if necessary. One month after the first application, the lesion was reduced in volume but appeared to be stable in color (Figure 1(b)); therefore, it was decided to perform another laser application. The patient reported the appearance of a slight purpura immediately after both laser sessions. At 12-month follow-up, a new ultrasound exam was performed: it showed a discreet volumetric reduction, with a microvacuolar structure with clear polycyclic edges, some fibrocalcific striae, and 2.2 mm calcifications of posterior profile (Figure 2(b)). The residual lesion size was 15.5 × 10.1 × 12 mm (Figure 1(c)). No similar adjacent malformations were recognized. At 24- and 36-month follow-up, the obtained outcomes remained clinically successful (Figure 1(d)). At 24-month imaging follow-up, the ultrasound exam showed a lesion with clear polycyclic edge with finely evident cleavage plane on contiguous uninjured tissue, not reflective structure with thin striae of fibrosis and vacuolar areoles, and 2 mm calcification of intense reflectivity (Figure 2(c)). Residual lesion was 14 × 7.9 × 9.7 mm with no-anarchic vessel arborization. In addition, 36 months after treatment, the imaging confirmed the persistence of a solid polylobulated lesion with clear polycyclic edge and striae of fibrosis of 14.4 × 6.7 mm. Color/power Doppler showed a single vascular pole afferent to the lesion, originating from the deep planes of the tongue, with physiological intralesional arborization. Therefore, the fibrotic structure underwent a peristructural injection of triamcinolone acetonide to be reduced. At 40 months, the malformation size was reduced and clinically not appreciable (Figures 1(e) and 2(d)).

## 3. Discussion

AVMs are considered the most difficult vascular malformation to be treated [17], and during recent years, several techniques were proposed with the main objective of destroying vascular nidus [4].

Principal approaches include embolization and surgery [4, 18]. These treatments might report some risks, and operators must be aware of complications; indeed, embolization, even if it is the first-choice therapy, could lead to local complication including mucosal ulcerations, nerve injury, tissue necrosis, and systemic complications such as cardiac toxicity and collapse [4, 18]. Accordingly, surgical resection is an invasive approach that might cause hemorrhagic complication [8]. It was the unique therapy proposed for years and now is often associated with embolization [4, 18]. This combination is indicated when the AVM is small and appears to be focal and completely resectable; however, if the removal may not be completely performed, relapse could be present [16].

More recently, laser treatment was proposed [16] to overcome the drawbacks of traditional approaches. The laser usually used to treat AVMs is the Nd:YAG with a fiber applied both superficially and percutaneously at the interstitial level [19]. However, to be more conservative and less traumatic as possible, dye laser might be proposed. In this light, to the best of our knowledge, this is the first study in which pulsed dye laser was applied as unique therapeutic strategy to treat AVMs. It is a highly selective wavelength of 595 nm that interacts with the oxyhemoglobin contained in the circulating blood and concentrates its power on red targets [18]. The red capillaries and vascular anomalies are coagulated without interesting the superficial layers of the tissue and are reabsorbed in a variable span of time depending on treated lesion [18]. Thanks to its selective action performed as photothermolysis, pulsed dye laser has been already used in treatment of superficial capillary malformations [16], but not yet applied for AVM therapy. In the case herein reported, the patient should undergo to a massive resection of the left hemitongue to solve AVM, resulting in a serious mutilation and a worsening of the quality of life. In addition, considering the great innervation of the tongue, the embolization might create nerve damage, causing dysesthesia. The obtained results not only demonstrated that laser treatment was accepted by the patient but also proved to be effective; indeed, vascular anarchic arborization forming the “nidus” was completely solved in 2 laser sessions, without any recurrence in 40-month follow-up. The patient did not have any discomfort during the laser applications and during the postoperative days, probably due to the minimal invasiveness of the therapy. Moreover, the technique was simple and manageable for clinician in terms of time and patient's compliance.

The success of this novel approach might be attributable to the clinical and morphological characteristics of the AVM reported within the present report: it involved the tongue in all its thickness, making possible to directly act on the lesion even with less penetration ability than Nd:YAG. Moreover, being the presented AVM classified as type IIIb of Cho score [4] and type II of Yakes score [5], it might be comparable to a capillary malformation. However, the preliminary and limited results provided by this case report should be further supported by treating deeper lesion with greater extension in a larger sample size.

## 4. Conclusion

Within the limitation of the present study, this case report suggested that dye laser might be used as a valid therapeutic option for the treatment of superficial mucosal AVM with a less invasive approach. Nonetheless, further research studies and clinical trials comparing different laser procedures should be carried out to elucidate which is the most reliable and best accepted treatment option.

## Data Availability

The data used to support the findings of this study are available from the corresponding author upon request.
